# The Influence of Silica Nanoparticles on Ionic Liquid Behavior: A Clear Difference between Adsorption and Confinement

**DOI:** 10.3390/ijms141021045

**Published:** 2013-10-18

**Authors:** Yaxing Wang, Cheng Li, Xiaojing Guo, Guozhong Wu

**Affiliations:** 1 Shanghai Institute of Applied Physics, Chinese Academy of Sciences, Shanghai 201800, China; E-Mails: wangyaxing@sinap.ac.cn (Y.W.); licheng@sinap.ac.cn (C.L.); guoxiaojing@sinap.ac.cn (X.G.); 2 Graduate University of Chinese Academy of Sciences, Beijing 100049, China

**Keywords:** confinement, adsorption, ionic liquid, melting point, phase behavior

## Abstract

The phase behaviors of ionic liquids (ILs) confined in nanospace and adsorbed on outer surface of nanoparticles are expected to be different from those of the bulk. Anomalous phase behaviors of room temperature ionic liquid tributylhexadecylphosphonium bromide (P_44416_Br) confined in ordered mesoporous silica nanoparticles with average pore size 3.7 nm and adsorbed on outer surface of the same silica nanoparticles were reported. It was revealed that the melting points (*T*_m_) of confined and adsorbed ILs depressed significantly in comparison with the bulk one. The *T*_m_ depressions for confined and adsorbed ILs are 8 °C and 14 °C, respectively. For comparison with the phase behavior of confined P_44416_Br, 1-butyl-3-methylimidazolium bromide (BmimBr) was entrapped within silica nanopores, we observed an enhancement of 50 °C in *T*_m_ under otherwise similar conditions. The XRD analysis indicates the formation of crystalline-like phase under confinement, in contrast to the amorphous phase in adsorbed IL. It was confirmed that the behavior of IL has clear difference. Moreover, the complex π-π stacking and H-bonding do not exist in the newly proposed phosphonium-based IL in comparison with the widely studied imidazolium-based IL. The opposite change in melting point of P_44416_Br@SiO_2_ and BmimBr@SiO_2_ indicates that the cationic species plays an important role in the variation of melting point.

## 1. Introduction

Room temperature ionic liquids (ILs), solely composed of organic cations and inorganic/organic anions of varying sizes, have received considerable attention in past years due to a wide range of applications and scientific research interests [1–3]. The tunable cations and anions lead to their peculiar chemical and physical properties, for example, negligible volatility, thermal stability, nonflammability, and high ionic conductivity and so forth. In recent years, many researchers focused on developing ILs for certain application and synthesizing a series of promising hybrid ionogel material [4–7], in which the ILs exist under the confinement or immobilization environment. As a matter of fact, several reports including theoretical and experimental work have found the anomalous phase behavior of ILs and other liquid confined in nanospace or immobilized on the surface of nanoparticles. The obvious difference between confined or adsorbed ILs and the bulk is the dramatic change in melting point (*T*_m_). Kanakubo *et al*. [8] found that *T*_m_ was depressed as 30 °C in comparison with the bulk when (Bmim)(CF_3_SO_3_) and (Bmim)((CF_3_SO_3_)_2_N) were confined within CPGs (controlled-pore glasses). Kim *et al*. [9] reported the melting point enhancement of imidazolium-based ILs confined in GMLs (graphene multilayers). Recently, our group found [10] that the interfacial effects and hydrogen-bonding networks between ILs and nano-SiO_x_ are responsible for the decrease of melting point of ILs. Moreover, we elucidated [11] that compressed gas within nanopores plays an important role in changing the melting point of confined ILs. The previous works have shown that nanoconfinement procedures as well as different nanoporous materials all contribute to the change in phase behavior of ILs. Furthermore, mesoporous materials with well-defined pore structures which could be easily synthesized and modified have great potential for separation, catalysis, semiconductive materials and so on [12]. Design for confined nanospaces incorporating with special guest molecules and atoms in metallocages and metal-organic frameworks also move on the development of functional materials [13,14].

With the combination of our continuous efforts to investigate the phase behavior of imidazolium-based ILs on the surface of mica [15], polystyrene submicrospheres [16], inside of multiwalled carbon nanotubes [17] and SiO_2_ nanopores [18], in this study, new proposed phosphonium-based IL P_44416_Br was filled into SiO_2_ nanopore under conditions of ultrahigh vacuum and adsorbed onto the outer surface of SiO_2_ by stirring the IL and SiO_2_ in a vessel, respectively. The difference between the newly proposed IL P_44416_Br and the widely used imidazolium-based IL is the distinct cationic species: the former consists of an atom phosphorus center and a hydrocarbon skeleton. Thus, the complex π-π stacking and H–bonding do not exist in P_44416_Br. For comparison, we also entrapped imidazolium-based IL BmimBr within the same type of SiO_2_ nanopores. Differential scanning calorimetry (DSC) was employed to detect the phase behaviors of confined and adsorbed ILs. We observed a significant *T*_m_ depression for both confined and adsorbed P_44416_Br, but the depression is more pronounced for the adsorbed one; however, the contrast experiment indicated the enhancement *T*_m_ of BmimBr confined in SiO_2_ nanopore. X-ray diffraction analysis was performed to investigate the property variation of ILs existing in two independent circumstances.

## 2. Results and Discussion

**Figure 1:**
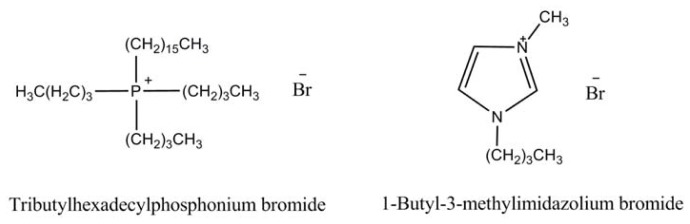
Two types of ionic liquids with various cations and the same anion.

**Figure 2:**
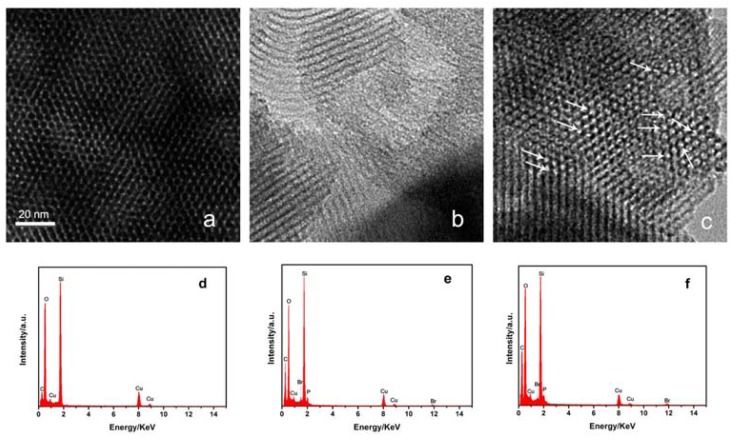
TEM images and the corresponding EDX spectra of SiO_2_ nanoparticles (**a**,**d**), P_44416_Br/SiO_2_ (**b**,**e**), and P_44416_Br@SiO_2_ (**c**,**f**). The scale bar in (**a**) applies to all three images. The arrows show the existence of ILs in nanopores.

The ionic liquids in this study have different cations and their models are shown in Figure 1. The new proposed phosphonium-based IL tributylhexadecylphosphonium bromide (P_44416_Br) is entrapped within ordered mesoporous SiO_2_ with pore size 3.7 nm at 100 °C under conditions of ultrahigh vacumm (1 × 10^−5^ Pa). By this procedure, as illustrated in our previous work [11], the air in the cavities of SiO_2_ particles could be completely removed and the IL could be easily entrapped in cavities of SiO_2_. The IL-filled samples will hereafter be referred to as P_44416_Br@SiO_2_. The TGA analysis shows that the loading amount of IL in P_44416_Br@SiO_2_ is 39 wt%. Surface adsorption experiment for P_44416_Br and SiO_2_ in a mass ratio of 39:100 was also carried out, and the sample was abbreviated to P_44416_Br/SiO_2_. Transmission electron microscopy (TEM) was conducted to characterize the morphology of SiO_2_, P_44416_Br/SiO_2_ and P_44416_Br@SiO_2_. As shown in Figure 2, the confined IL in the P_44416_Br@SiO_2_ reveals clearly that some IL is entrapped in the channel of SiO_2_ (as indicated by arrows in Figure 2c), while the surface adsorption experiment shows that the SiO_2_ is covered with IL (as shown in Figure 2b). The morphology of confined IL is consistent with the work by Ji *et al*. [19], who reported the tungsten carbide confined in the channels of SBA-15 mesoporous silica.

**Figure 3:**
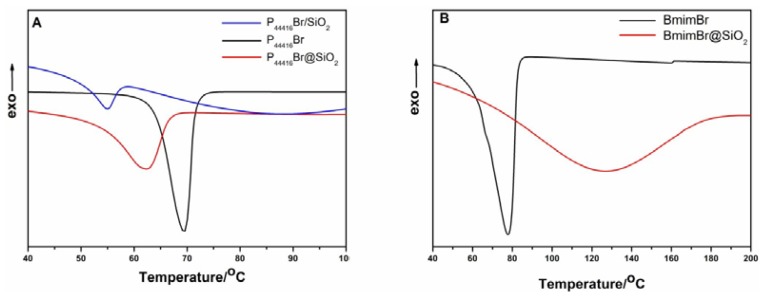
Differential scanning calorimetry (DSC) heating curves for (**A**) P_44416_Br/SiO_2_; P_44416_Br; P_44416_Br@SiO_2_; (**B**) BmimBr; BmimBr@SiO_2_.

To investigate the phase behavior change of ILs, DSC experiments were carried out. Figure 3A shows DSC curves for P_44416_Br/SiO_2_, bulk P_44416_Br, and P_44416_Br@SiO_2_. The melting point of P_44416_Br/SiO_2_ is observed to be 55 °C, being 14 °C lower than that of the bulk P_44416_Br. Our result is in good agreement with the literature [20] in which a remarkable depression *T*_m_ is also observed when imidazolium-based IL is immobilized on the surface of SiO_2_. The reduction of *T*_m_ in our results is mainly attributed to the interaction between IL and SiO_2_. As illustrated in the literature [21], the intermolecular interactions between IL and surface will decrease the mobility of cations near the interface, the cations trend to absorb onto the surface, and thus the cations are trapped in a higher entropic state, leading to a depression in *T*_m_[22]. The H–bonding between IL and hydroxyl of surface will possibly lead to an increase in *T*_m_, but the overall effect observed is the depression in *T*_m_. However, a novel depression in melting point of confined ILs was observed when P_44416_Br was entrapped in SiO_2_. The melting point of P_44416_Br@SiO_2_ is 61 °C, which is 8 °C lower than that of pristine P_44416_Br (Figure 3A). As reported in previous works [9,17,18], the nanoconfinement can lead to the enhancement of *T*_m_, while, in this case, the depression in *T*_m_ of confined ILs P_44416_Br is mostly due to the choice of different cationic species. For comparison, imidazolium-based IL BmimBr was entrapped within SiO_2_, an elevation of *T*_m_ was observed (Figure 3B). In nanoconfinement, IL will be compressed leading to a reduction in distance between anion and cation [18]. We supposed that the reduction in distance between anion and cation will result in the compression of C–H···Br hydrogen bonding that exists in imidazolium-based IL, this as well as strengthened coulomb force resulting from compression play important roles leading to an increased melting point of confined ILs. Konstantin [23] also reported an increased melting point originating from the presence of extremely short C–H···F contacts in the crystal and atomistic molecular dynamics simulation illustrated that imidazolium-based IL formed the dramatic hydrogen-bonded network structure under confinement [24,25]. The compression of confined IL ought to be a general phenomenon. In sample P_44416_Br@SiO_2_ the compression will strengthen the coulomb force and enhance the melting point of confined IL, the absence of C–H···Br hydrogen bonding and the IL-wall interaction should all contribute to the change in melting point; thus, the integrative effects depressed the melting point. This is the reason that the melting point of P_44416_Br@SiO_2_ is higher than that of P_44416_Br/SiO_2_. In other words, the cationic species, which would result in the different interaction between cation and anion, plays a non-negligible role in changing the melting point of confined ILs.

**Figure 4:**
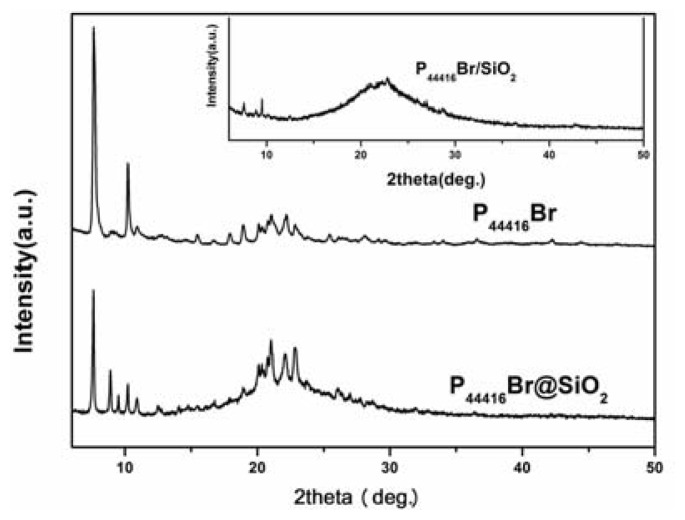
XRD patterns of the bulk P_44416_Br and the sample P_44416_Br @SiO_2_. Inset: XRD pattern of sample P_44416_Br/SiO_2_.

**Scheme 1:**
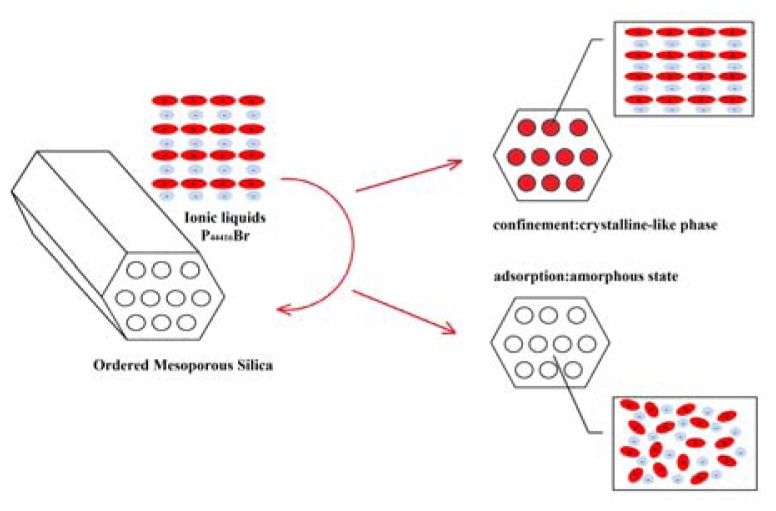
Schematic diagram of the influence of silica nanoparticles on ionic liquid behavior.

It is generally believed that the confinement effect can lead to the structural vibration of IL. Molecular dynamics simulations reported that 1,3-dimethylimidazolium chloride undergoes a liquid-to-solid transition under the confinement of graphite walls and 1-butyl-3-methylimidazolium hexafluorophosphate possesses long-range crystalline order at 300 K in carbon nanopore [26,27]. To further understand the structural variation of confined and adsorbed ILs, the X-ray diffraction measurement was employed. Figure 4A implies the formation of crystalline state of the confined ILs, some new peaks appear in comparison with the bulk P_44416_Br. However, as illustrated in Figure 4B, the P_44416_Br/SiO_2_ is speculated to be amorphous, the wide-bound peak between 15° and 30° is due to the amorphous peak of SiO_2_. It is noted that the bulk P_44416_Br has crystal-like structure and the XRD analysis indicates that the conformation or stacking of ILs under confinement or adsorption has critical change. The influence of silica nanoparticles on the behavior of P_44416_Br is further proved from the enthalpy of fusion for bulk P_44416_Br, P_44416_Br@SiO_2_ and P_44416_Br/SiO_2_, shown in Table 1. Different enthalpy of fusions between P_44416_Br@SiO_2_ and P_44416_Br/SiO_2_ indicate the difference in structure for confined and adsorbed ILs. The interfacial effect and hydroxyl of surface most likely disarrange the structure of the adsorbed IL and confinement may result in “compact stacking” (Scheme 1).

## 3. Experimental Section

### 3.1. Materials

P_44416_Br, BmimBr ionic liquids, silica nanoparticles and methanol were obtained from Sigma-Aldrich^®^ (Shanghai, China). The average pore diameter of silica is 3.7 nm. All the materials were reagent grade and used as purchased.

### 3.2. Experimental Details

Exactly 214.6 mg SiO_2_ was put into a two-necked flask (one of the necks was connect with a high-vacuum line and the others was sealed). The flask was heated 100 °C for 6 h under vacuum (1 × 10^−5^ Pa) to remove the gas and water in the cavities of SiO_2_ nanopores. Then 436.2 mg P_44416_Br ionic liquids dissolved in methanol was transferred into the flask through a syringe and the mixture was subsequently heated at 100 °C for 6 h. The P_44416_Br ionic liquid filled into the cavities of SiO_2_. The as-prepared product was cooled to room temperature and washed with methanol to remove the P_44416_Br adsorbed on the outer surface of SiO_2_ and then dried at 50 °C in an oven for 12 h to remove any volatiles to obtain the final sample P_44416_Br@SiO_2_. The same procedure was employed for the preparation of BmimBr@SiO_2_.

Exactly 430.3 mg SiO_2_ and 168.2 mg P_44416_Br ionic liquid (mass ratio = 100:39) were added to an appropriate amount of methanol solution in a glass beaker. The solution was stirred for 24 h and then dried at 50 °C in an oven for 12 h to remove any volatiles to obtain the final sample P_44416_Br/SiO_2_.

### 3.3. Characterizations

Phase behaviors of the bulk P_44416_Br, the sample P_44416_Br@SiO_2_ and P_44416_Br/SiO_2_ were determined by differential scanning calorimetry (DSC-822e, Mettler-Toledo Corp.). The programmed heating rate is 10 °C/min, the sample about 12 mg was placed in an aluminum pans with pierced lids.

X-ray diffraction (XRD) measurement patterns were recorded with a Philips X-ray diffractometer (PW-1710) using Cu K_α_ radiation ranging from 5° to 50°.

Transmission electron microscopy (TEM) was performed on a Zeiss EM 912 operated at 120 kV.

## 4. Conclusions

In summary, we studied the phase behavior of phosphonium-based IL P_44416_Br in two independent circumstances (adsorption and confinement). We observed a remarkable depression in *T*_m_, the melting point of adsorbed IL, P_44416_Br/SiO_2_, was lower than that of confined IL, P_44416_Br@SiO_2_, and all two samples have a depressed melting point in comparison with the bulk one. The XRD measurement further illustrated different structural variation of P_44416_Br in two independent circumstances. In the context of previous work and the phase behavior of imidazolium-based IL confined in silica, BmimBr@SiO_2_, our results indicated that the cationic species plays an important role in changing the melting point. It was assumed that the interfacial effect and hydroxyl of surface are responsible for depression in melting point of the adsorbed P_44416_Br and that the confinement effect and interaction of IL contributed to the change in the melting point of confined P_44416_Br.

## Figures and Tables

**Table 1:** Enthalpy of fusion for bulk P_44416_Br, P_44416_Br@SiO_2_ and P_44416_Br/SiO_2_.

	P_44416_Br	P_44416_Br@SiO_2_	P_44416_Br/SiO_2_
Enthalpy of fusion/J·g^−1^	73.02	21.67	17.90
